# Editorial: New Aspects in Hypogonadism

**DOI:** 10.3389/fendo.2020.00426

**Published:** 2020-06-30

**Authors:** Andrew A. Dwyer, Richard Quinton

**Affiliations:** ^1^Boston College, William F. Connell School of Nursing, Chestnut Hill, MA, United States; ^2^Massachusetts General Hospital, Reproductive Endocrine Unit, Boston, MA, United States; ^3^Newcastle-upon-Tyne Hospitals Foundation NHS Trust (Royal Victoria Infirmary) & Institute of Genetic Medicine, University of Newcastle-upon-Tyne, Newcastle-upon-Tyne, United Kingdom

**Keywords:** Kallmann syndrome, Klinefelter syndrome, hormone therapy, anemia, minipuberty, aging

Testosterone and estradiol play critical, wide-ranging roles during development, before and beyond the traditional notion of a reproductive lifespan. In fetal life, testosterone is crucial for sexual differentiation and the proper development of male external genitalia (1). During late pregnancy and the first 6 months of neonatal life (i.e., minipuberty), testosterone and follicle-stimulating hormone play a major role in priming the male reproductive axis for fertility potential in later life (2). During puberty, rising sex steroids induce the development of secondary sexual characteristics—reaching adult normal levels necessary for full reproductive capacity. Later in life, bone and metabolic health are affected by falling estrogen levels of menopause and progressive declines in circulating testosterone levels in men (3, 4).

Hypogonadism describes the deficiency of sex steroids and accompanying symptoms. Classically, hypogonadism has been divided into primary (gonadal failure) and secondary (neuroendocrine defects). Given the role of sex steroids in development, hypogonadism may have far-reaching consequences on health and well-being including absent minipuberty, incomplete sexual maturation, altered sexual behavior, infertility, and disrupted metabolism. There is a broad array of unanswered questions about hypogonadism. For example, Klinefelter syndrome has a highly varied phenotype, presumably due to differential silencing of the supernumerary X, yet we know very little about the causality. Further, questions remain regarding the optimal timing of introducing testosterone replacement and fertility preservation to improve metabolic health in adulthood and improved quality of life. Similarly, the molecular basis of secondary hypogonadism (e.g., congenital hypogonadotropic hypogonadism/Kallmann syndrome) has been charted and absent minipuberty and cryptorchidism are known to be an early life determinants of fertility potential. Currently, it remains unclear if neonatal gonadotropin therapy in these contexts may improve future fertility, although evidence that it may reduce the need for surgical orchidopexy is reasonably compelling. Despite the availability of effective treatment for decades, there is yet consensus on the optimal sex steroid replacement regimen and approach for treating adolescent males and females with hypogonadism. Late onset hypogonadism has gained increasing interest, yet the etiology remains to be fully elucidated, the degree of testosterone deficiency is often quite modest and longer term clinical consequences remain uncertain. Moreover, the definition itself may need to be revised in line with more recent data, so as to specifically reference aging-related primary hypogonadism. The nine articles in this Research Topic shed light on diverse, neglected, but nevertheless important and illuminating aspects of hypogonadism.

Two articles address key—but often overlooked—principles and applications of sex steroid treatment in hypogonadal men and women. Al-Sharefi et al. concisely summarize the evidence on androgens and anemia, highlighting the clinical relevance of testosterone for men with chronic kidney disease, or with apparently unexplained anemia-of-aging. A second article examines the evidence relating to what best constitutes safe and adequate estrogen replacement for transgender women and young women with premature ovarian failure. Authors draw lessons that deserve wider application to the care of hypogonadal women. Specifically, they identify the advantages of using native 17β-estradiol (compared to ethinylestradiol or conjugated equine estrogen), and the logic of adjusting treatment doses according to serum estradiol levels as well as patient symptoms and bone densitometry (Swee et al.).

Two further articles examine the early life impact of hypogonadism. Kuiri-Hänninen et al. review activation of the hypothalamic-pituitary-testicular axis in relation to testicular descent. Authors draw attention to minipuberty for providing a window of opportunity to examine the axis in early infancy (Kuiri-Hänninen et al.). The second article provides a complementary perspective underscoring that minipuberty is an unique opportunity for early diagnosis of hypogonadism and timely initiation of treatment to improve health, fertility and well-being (Swee and Quinton). Two review articles provide comprehensive pictures of the psychosocial impact of primary and secondary hypogonadism in males. Hanna et al. present a narrative review combining quantitative and qualitative studies on Klinefelter syndrome to provide a holistic view of how primary hypogonadism can affect psychological and emotional well-being. The complementary article on psychological aspects of secondary hypogonadism is co-authored by patient leader, facilitator and educator, Neil Smith. This article provides a comprehensive review of the state of the science in the area, supplemented by patient perspectives that are too-often neglected in the medical literature (Dwyer et al.).

A controversial topic of debate concerns late-onset hypogonadism—sometimes called andropause or low T syndrome by enthusiast clinicians. Swee and Gan review evidence from recent population-based and intervention studies providing a balanced overview of the diagnosis, pathophysiology, and management approaches. Finally, two articles provide insights into the mechanisms underlying the metabolic effects of hypogonadism and patho-mechanisms contributing to decreased testosterone production with aging. Dimakopoulou et al. summarize evidence from animal models to identify the links between metabolism and reproduction—with particular attention to diabetes mellitus as a predisposition to male hypogonadism. Lee et al. present mouse data on the role of nitroso-redox imbalance in decreased testosterone production. Such mechanistic studies deepen our understanding of strong association between hypogonadism and diabetes and patho-mechanisms underlying late-onset hypogonadism. It is striking how the ~3-fold greater risk of having Type 2 diabetes mellitus observed in Klinefelter syndrome also seems to be common to other forms of primary gonadal insufficiency in males, including the age-related form.

The articles included in this Research Topic fill important gaps in current understanding of hypogonadism. Articles cover the human lifespan, from late intrauterine life and minipuberty through to old age. Contributions include perspectives from basic scientists, clinicians, psychologists, sociologists, and patients advocates. We hope the diverse articles included in this Research Topic will spark reflection on hypogonadism and a deeper appreciation for some of these neglected areas of inquiry.

## Author Contributions

AD made substantial contributions to the design of the work, drafted the editorial and approved the final version. RQ made substantial contributions to the design of the work, edited the manuscript, and approved the final version. All authors contributed to the article and approved the submitted version.

## Conflict of Interest

The authors declare that the research was conducted in the absence of any commercial or financial relationships that could be construed as a potential conflict of interest.
